# Supplementary material for: Identification of photoperiod-induced specific miRNAs in the adrenal glands of Sunite sheep (Ovis aries)
Source: Front Vet Sci. 2022 Jul 22;9:888207. doi: 10.3389/fvets.2022.888207 (PMC9354845; doi:10.3389/fvets.2022.888207)
Supplement: Supplementary file 1 [file Data_Sheet_1.ZIP › Supplementary file/miRDeep2 file.zip!/miRDeep2 file/Novel_Structure/SP42A2/Novel_610.pdf]

| | | |
|---|---|---|
| Provisional ID | : | NC_019484.2_43715 |
| Score total | : | 0.5 |
| Score for star read(s) | : | -1.3 |
| Score for read counts | : | 0 |
| Score for mfe | : | 0.2 |
| Score for randfold | : | 1.6 |
| Score for cons. seed | : | |
| Total read count | : | 77 |
| Mature read count | : | 77 |
| Loop read count | : | 0 |
| Star read count | : | 0 |

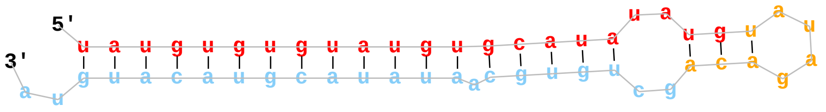


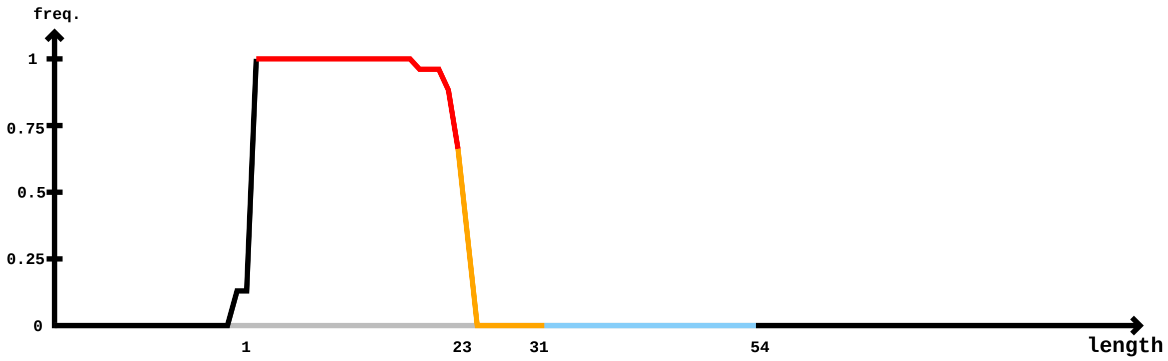


**Mature** **Star**

```
5'- uauuaugaggauuucuauauauguguguaugugcauauauguauagacagcugugcaauauacguacauguauguauacaaaugucauuggugugcauuagguaacuucuc   -3'    exp
    ......((((...((((((((((((((((((((((((..(((....)))..))))).)))))))))))))))(((((((.(((...))).))))))).))))...))))..     reads     mm     sample
    .................uauauguguguaugugUau...........................................................................     3         1      seq
    .................uauauguguguaugugUauaua........................................................................     6         1      seq
    .................uauauguguguaugugUauauau.......................................................................     1         1      seq
    ...................uauguguguaugugUauauau.......................................................................     16        1      seq
    ...................uauguguguaugugUauauaug......................................................................     50        1      seq
    ...................uauguguguaugugUauauaugu.....................................................................     1         1      seq
```
